# CRISPR/Cas-based screening of long non-coding RNAs (lncRNAs) in macrophages with an NF-κB reporter

**DOI:** 10.1074/jbc.M117.799155

**Published:** 2017-10-19

**Authors:** Sergio Covarrubias, Elektra K. Robinson, Barbara Shapleigh, Apple Vollmers, Sol Katzman, Nicole Hanley, Nicholas Fong, Michael T. McManus, Susan Carpenter

**Affiliations:** From the ‡ Department of Molecular, Cell, and Developmental Biology and; ¶ Center for Biomolecular Science and Engineering, University of California, Santa Cruz, California 95064 and; the § Department of Microbiology and Immunology, Diabetes Center, University of California San Francisco, San Francisco, California 94143

**Keywords:** CRISPR/Cas, inflammation, long non-coding RNA (long ncRNA, lncRNA), macrophage, NF-κB (NF-κB), toll-like receptor (TLR)

## Abstract

The innate immune system protects against infections by initiating an inducible inflammatory response. NF-κB is one of the critical transcription factors controlling this complex response, but some aspects of its regulation remain unclear. For example, although long non-coding RNAs (lncRNAs) have been shown to critically regulate gene expression, only a fraction of these have been functionally characterized, and the extent to which lncRNAs control NF-κB expression is unknown. Here, we describe the generation of a GFP-based NF-κB reporter system in immortalized murine bone marrow–derived macrophages (iBMDM). Activation of this reporter, using Toll-like receptor ligands, resulted in GFP expression, which could be monitored by flow cytometry. We also established a CRISPR/Cas9 gene deletion system in this NF-κB reporter line, enabling us to screen for genes that regulate NF-κB signaling. Our deletion-based approach identified two long intergenic non-coding(linc)RNAs, lincRNA-Cox2 and lincRNA-AK170409, that control NF-κB signaling. We demonstrate a potential novel role for lincRNA-Cox2 in promoting IκBα degradation in the cytoplasm. For lincRNA-AK170409, we provide evidence that this nuclearly-localized lincRNA regulates a number of inflammation-related genes. In conclusion, we have established an NF-κB–GFP iBMDM reporter cell line and a line that stably expresses Cas9. Our approach enabled the identification of lincRNA-Cox2 and lincRNA-AK170409 as NF-κB regulators, and this tool will be useful for identifying additional genes involved in regulating this transcription factor critical for immune function.

## Introduction

It is now appreciated that the majority of the human genome (85%) is actively transcribed, yet less than 3% encodes for protein-coding genes (1). lncRNAs^2^ represent the largest group of RNAs produced from the genome and are defined as transcripts greater than 200 nucleotides in length, lacking protein-coding capacity (2). GENCODE version 24 has ∼16,000 annotated lncRNAs within the human genome, yet only 1% of these has been functionally characterized. From those that have been characterized, it is clear that lncRNAs are able to function through a variety of mechanisms to regulate gene expression (3).

Macrophages are important innate immune cells that function to recognize and defend against pathogens, while also helping to promote development of effective adaptive immune responses (4). Membrane-bound Toll-like receptors (TLRs) play a critical role in recognizing specific pathogen-associated molecular patterns initiating a complex inflammatory signaling cascade culminating in the activation of transcription factors, including NF-κB (5). NF-κB, in turn, specifically activates numerous inflammatory genes, which function to provide protection against invading microbes. Although NF-κB activation is critical for eliminating pathogens, excess activation of this pathway can result in a variety of inflammatory-based diseases.

CRISPR/Cas9 technology has revolutionized the field of functional genomics by providing a novel tool for interrogating gene function. CRISPR/Cas9 is a deoxyribonuclease (DNase) that can be specifically targeted to genomic regions via a guide RNA (gRNA) (6, 7). Targeting of Cas9 to a region results in a blunt double-stranded DNA break yielding small deletions in the repaired sequence (8). Small deletions are appropriate for disrupting open reading frames (ORFs) of protein-coding genes but are insufficient for disrupting lncRNAs that can be tens of thousands of kilobases in length (9). Using 5′- and 3′-flanking gRNAs, large stretches of DNA can be deleted, allowing for efficient disruption of lncRNA-encoding loci (10).

Here, we describe a novel NF-κB–GFP reporter system in murine macrophages combined with CRISPR/Cas9 capabilities to allow robust interrogation of novel lncRNAs involved in TLR/NF-κB signaling. We utilize Cas9-mediated deletion of candidate lncRNA loci and characterize two lncRNAs, lincRNA-Cox2 and lincRNA-AK170409, and demonstrate their role in NF-κB signaling. We show that although both lincRNAs alter NF-κB signaling, they likely function in distinct ways. We provide evidence that lincRNA-Cox2 affects IκBα degradation in the cytoplasm, whereas nucleus-restricted lincRNA-AK170409 regulates numerous inflammation-related genes by modulating other steps of NF-κB activation. Here, we established an NF-κB–GFP iBMDM reporter cell line with Cas9 capabilities, allowing for rapid screening of novel regulators of the NF-κB pathway. Our characterization of lincRNA-Cox2 and lincRNA-AK170409 serves as an example of how screening-based identification of novel genes can lead to additional mechanistic understanding of ways to regulate NF-κB.

## Results

### Development of an NF-κB–GFP reporter system in murine macrophages

**Figure 1.**
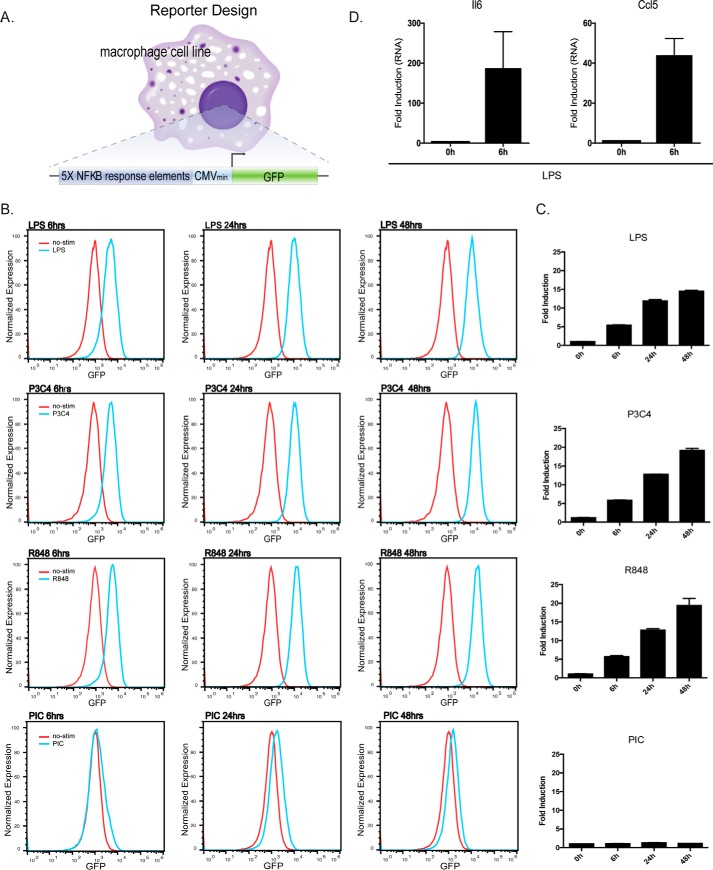
**NF-κB–GFP reporter macrophages can respond to various Tlr ligands.** *A,* immortalized BMDMs were infected with the NF-κB reporter construct containing 5× NF-κB-binding sites upstream of a minimal promoter GFP. *B,* iBMDMs were either not stimulated (*red*) or stimulated with LPS (Tlr4), Pam3CSK4 (Tlr1/2), R848 (Tlr7/8), or poly(I:C) (Tlr3) ligands (*blue*) for the indicated hours. At the indicated times, cells were analyzed by FACS to assess induced GFP levels. *C,* histograms from *B* are summarized in *bar format. D, Il6* and *Ccl5* mRNA levels were assessed by qPCR via normalization to the housekeeping gene *Gapdh*. Results are presented as mean ± S.D. for at least three replicate experiments.

Numerous fluorescence and chemiluminescence-based NF-κB reporters have been developed in a variety of cell lines (11, 12), which have facilitated the identification of novel genes involved in regulating NF-κB activity. Until recently (13), no fluorescent-based NF-κB reporter systems had been described in bone marrow–derived macrophages (BMDMs). BMDMs are critical mediators of the inflammatory response and represent a more physiologically relevant cell line compared with other reporters currently available. We constructed a GFP-based NF-κB reporter system by adding 5× NF-κB-binding motifs (GGGAATTTCC) upstream of the minimal CMV promoter-driven GFP (Fig. 1*A*). Immortalized BMDMs (iBMDMs) were lentivirally infected and clonally selected for optimal reporter activity. We tested the utility of our system by stimulating cells with various TLR ligands for 6, 24, or 48 h (Fig. 1, *B* and *C*). Stimulated cells showed >10-fold induction of GFP for LPS (Tlr4), Pam3CSK4 (Tlr1/2), and R848 (Tlr7/8), while showing weak induction with poly(I:C) (Tlr3) ligand (Fig. 1, *B* and *C*). We confirmed that the NF-κB reporter cells were capable of inducing endogenous proinflammatory genes *Il6* and *Ccl5* (Fig. 1*D*). Together, these data show that we have developed a functional GFP-based NF-κB reporter system in macrophages.

### Demonstration of CRISPR/Cas9 functionality in the NF-κB–GFP cells

**Figure 2.**
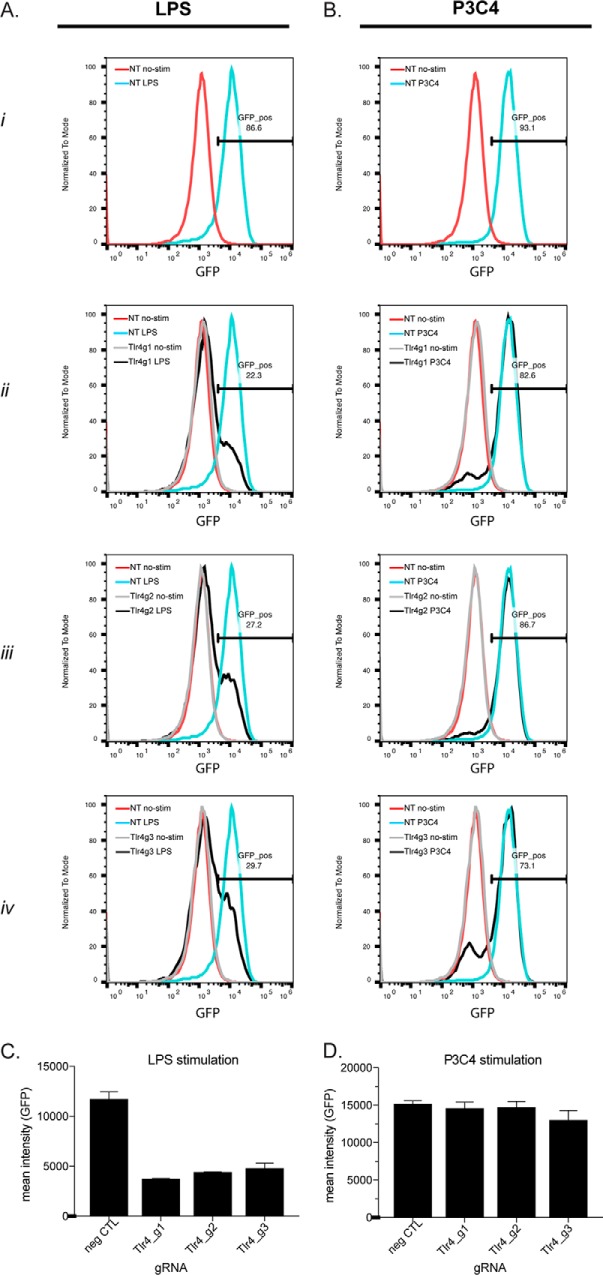
**Validation of CRISPR/Cas9 system functionality in NF-κB–GFP reporter macrophages.** iBMDM NF-κB–GFP-Cas9 cells were infected with anti-*Tlr4* gRNAs or negative control gRNA lentivirus and puro-selected to obtain pure gRNA-expressing cells. *A* and *B*, after 24 h of no-stimulation (*red*) or stimulation with either LPS (*A*) or Pam3CSK4 (*B*) (*blue*), GFP induction was assessed by FACS. *Row i* represents cells infected with NT, stimulated (*blue*) or no stimulation (*red*), and *rows ii–iv* show *Tlr4g1, Tlr4g2*, or *Tlr4g3*, respectively, stimulated (*black*) or no-stimulation (*gray*). *C* and *D*, histogram data from *A* and *B* are summarized in *bar format* for either LPS (*C*) or P3C4 (*D*) stimulation. Results are presented as mean ± S.D. from duplicate experiments.

One the most useful features of NF-κB reporter systems has been the ease of use for screen-based identification of genes that regulate the NF-κB pathway (14). CRISPR/Cas9 technology has revolutionized the field of functional genomics by providing a novel tool for interrogating gene function. We reconstituted the CRISPR/Cas9 system into our reporter cells using lentiviral transduction, allowing a versatile and easy way to interrogate novel gene function (supplemental Fig. S1*A*). Cas9 has been observed to have unwanted toxicity in cells (15). Nevertheless, we confirmed that Cas9 expression did not alter the function of our NF-κB reporter system (supplemental Fig. S1). To validate the CRISPR/Cas9 system, we cloned three gRNAs targeting *Tlr4* or non-targeting (NT) guides into a U6-gRNA lentiviral construct and infected Cas9-expressing iBMDM cells for 7 days to allow for complete editing of the target genes. LPS stimulation of the NT-expressing cells resulted in >10-fold induction of GFP as expected (Fig. 2*A*, *row i, blue*). In contrast, anti-*Tlr4* guide-expressing cells were not able to induce GFP upon LPS stimulation (Fig. 2*A*, *rows ii–iv, black*). We confirmed the specificity of the anti-*Tlr4* guides by demonstrating that Tlr2 stimulation was not altered (Fig. 2*B*, *rows ii–iv, black*). Quantifications of mean fluorescence intensities for two representative experiments are shown in Fig. 2, *C* (LPS) and *D* (P3C4). Together, these data demonstrate that we have successfully constructed an NF-κB reporter system in iBMDMs with functional Cas9 capabilities.

### Using deletion-based screening to identify lncRNAs important in regulating NF-κB

We have previously performed RNA sequencing on murine iBMDMs following stimulation with the Tlr1/2 ligand Pam3CSK4, and we identified 64 up-regulated lncRNAs following stimulation (16). From the data set, 20 of the lncRNAs were intergenic, meaning they possessed their own promoters and did not overlap any protein-coding genes, which allowed for deletion of loci without potential disruptions to neighboring coding genes. To select candidates for deletion, we focused on the highly up-regulated lincRNAs that overlap both our data (16), as well as the dataset generated by Lam *et al.* (17) where they stimulated cells with Pam3CSK4 and LPS. From these data, we focused our attention on lincRNA-Cox2 and lincRNA-AK170409. The expression profile of these lncRNAs supported that they were likely regulated by the NF-κB via TLR-signaling pathway; however, whether these lncRNAs were involved in regulating NF-κB has not been investigated.

**Figure 3.**
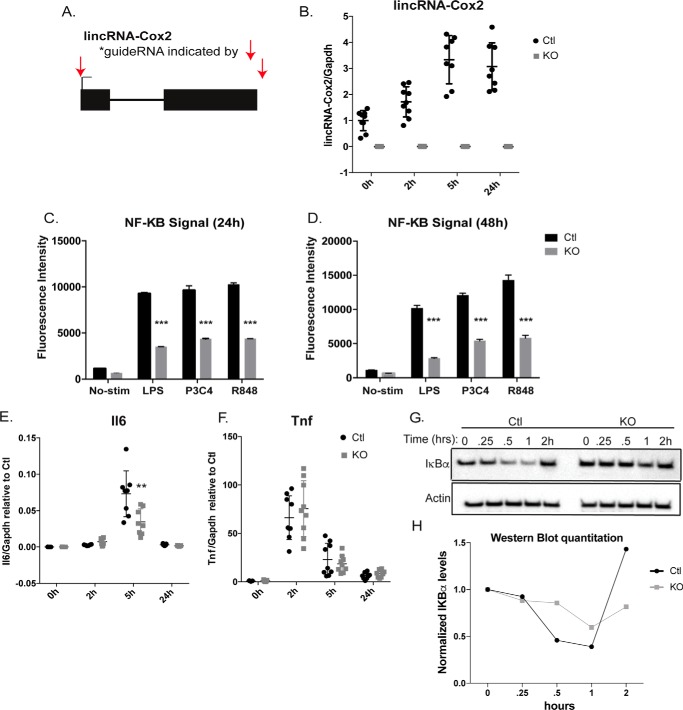
**Deletion of lincRNA-Cox2 disrupts NF-κB signaling.** *A,* schematic shows location of targeting by two gRNAs (*red arrow*), targeting upstream and downstream of the lincRNA-Cox2 locus. *B,* expression levels of lincRNA-Cox2 were assessed for control and KO cells by qPCR for indicated time points. *C* and *D*, mean fluorescence induction of GFP was assessed by FACS for control and KO BMDMs after LPS, P3C4, or R848 stimulation for 24 h (*C*) or 48 h (*D*). *E* and *F,* qPCR was used to assess mRNA expression levels for *Il6* (*E*) and *Tnf* (*F*) for the indicated time points. *G,* Western blot analysis for IκBα was performed for control (Ctl) and KO for indicated time points following LPS stimulation. *H,* quantitations of the Western blot from *G* are shown. All qPCR data were performed in biological triplicates and normalized to *Gapdh*. *, *p* < 0.05; **, *p* < 0.01; ***, *p* < 0.005.

**Figure 4.**
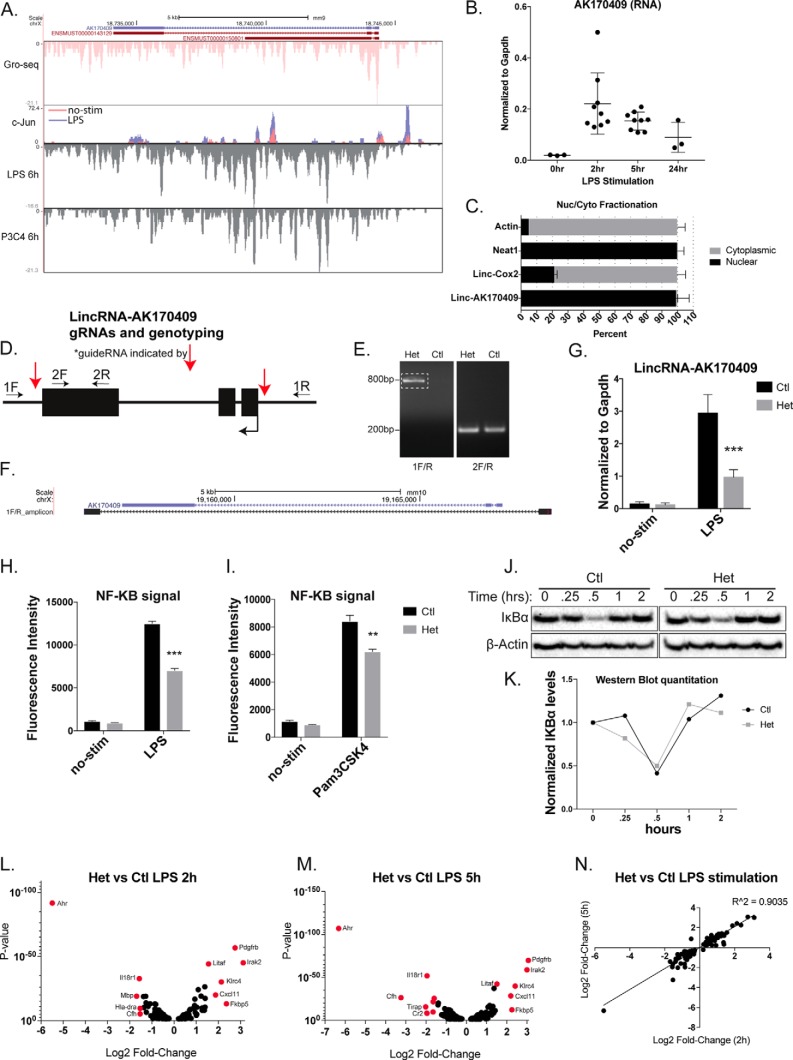
**Deletion of a novel lincRNA locus results in disruption of NF-κB signaling.** *A,* UCSC genome browser tracks showing the lincRNA-AK170409 locus, Gro-seq data, c-Jun transcription factor binding, and transcriptional induction of the locus by LPS and P3CSK4 ligands. *B,* expression levels of lincRNA-AK170409 were assessed following stimulation with LPS for the indicated hours (*hrs*). *C,* nuclear/cytoplasmic subcellular fractionation of LPS-stimulated cells was performed, RNA was extracted, and qPCR was done for the indicated genes. Actin (*Actb*) mRNA is a cytosol-localized control, whereas *Neat1* serves as a nuclearly-localized control. *D,* schematic shows location of targeting by two gRNAs (*red arrow*), targeting upstream and downstream of the AK170409 locus. Binding position of genotyping primers is shown for primer sets 1 and 2. *E*, amplicons for primer sets 1 and 2 were resolved on an agarose gel. Primer set 1 amplicon, only produced from AK170409 deleted alleles (*white dash line*), was sequenced and aligned to the mouse genome in *F. G,* expression levels of AK170409 were assessed for Ctl and heterozygote (*Het*) cells by qPCR following stimulation with LPS. *H* and *I*, mean fluorescence induction of GFP was assessed by FACS for Ctl and Het cells after 24 h of stimulation with LPS (*H*) or Pam3CSK4 (*I*). *J,* Western blot analysis for IκBα was performed for Ctl and Het cells for the indicated hours (*hrs*) post-LPS stimulation. *K,* quantitation of the Western blot from *J* are shown. *L* and *M, volcano plots* display differentially expressed genes in Het *versus* Ctl iBMDM stimulated with LPS for 2 h (*L*) or 5 h (*M*). *N,* mean fold changes for the 2 and 5 h were plot against each other. *R*^2^ value is calculated. All qPCR data were performed in biological triplicates and normalized to the housekeeping gene Gapdh. *, *p* < 0.05; **, *p* < 0.01; ***, *p* < 0.005.

We had previously established a role for lincRNA-Cox2 in regulating immune response genes (16). Knocking down lincRNA-Cox2 in murine iBMDMs using shRNA led to a reduction in expression of proinflammatory genes such as *Il6* (16), although the mechanism by which lincRNA-Cox2 regulates these genes remains unclear. Here, we delete the lincRNA-Cox2 locus using CRISPR/Cas9 to directly test the impact on NF-κB signaling. We designed two gRNAs targeting both 5′- and 3′-sequences flanking the lincRNA-Cox2 locus (Fig. 3*A*, *red arrows*). We isolated a clone containing a deletion in the locus and verified the loss of lincRNA-Cox2 expression by qPCR (Fig. 3*B*). We have previously shown that expression of lincRNA-Cox2 is most highly induced by TLR stimulation peaking at 5 h, and here we confirm this is also true in our iBMDM reporter system (Fig. 3*B*). We have also previously reported that lincRNA-Cox2 is directly regulated by ΝF-κB binding (17). A small number of NF-κB-induced lncRNAs have been demonstrated to function as positive/negative feedback regulators of NF-κB signaling (3). It was unclear whether lincRNA-Cox2 played this type of role. In this study, we found that disruption of the lincRNA-Cox2 locus leads to an overall decrease in NF-κB activity when cells were stimulated with a variety of ligands at both 24 h (Fig. 3*C*) and 48 h (Fig. 3*D*) post-stimulation. Induction of Il6 was diminished in the KO cells at the 5-h time point (Fig. 4*E*), whereas Tnf, which is most highly induced after 2 h of stimulation, was not altered (Fig. 4*F*). These data are consistent with our previous study using shRNA where we showed that lincRNA-Cox2 can affect genes at the level of transcription (16). Inducible activation of NF-κB depends upon proteosomal degradation of the inhibitor of NF-κB proteins (IκBs), which retains inactive NF-κB dimers in the cytosol (18). Surprisingly, we found that there was a dramatic decrease in IκBα degradation following 0.5 h of stimulation with LPS in the lincRNA-Cox2 KO cells (Fig. 3, *G* and *H*). The NF-κB subunit p65 has been shown to directly regulate IκBα levels (19). Hence, one explanation for the deficiency in IκBα degradation could be explained by alterations in p65 levels (19). We measured p65 RNA levels by qPCR in the control and KO cells and found no difference at the basal levels (supplemental Fig. S2), although there was a decrease in p65 levels at 5 h post-stimulation in the KO cells (supplemental Fig. S2). IκBα is localized to the cytoplasm, whereas lincRNA-Cox2 has been shown to be localized to both the nucleus and cytoplasm (Fig. 4*C*) (16). Our data support a possible role for lincRNA-Cox2 in the cytoplasm regulating the degradation of IκBα. IκBα degradation occurred at 0.5 h post-stimulation, which suggests that basal cytoplasmic expression of lincRNA-Cox2 could impact the NF-κB pathway; however, the mechanism of action remains to be determined. Here, we have established the utility of our NF-κB–Cas9 system for deletion-based screening for lncRNAs involved in NF-κB signaling.

### lincRNA-AK170409 is a novel regulator of NF-κB signaling

Given that a only a small number of NF-κB-induced lncRNAs have been demonstrated to function as positive and negative feedback regulators of NF-κB signaling, we reasoned that there could be other novel regulators of this pathway (3). We targeted the intergenic lincRNA-AK170409 because it showed high induction with LPS and Pam3CSK4 in both primary macrophages and in our iBMDMs reporter cells (Fig. 4*A*, *LPS* and *P3C4*). Additionally, this lincRNA possesses an inducible ChIP peak for transcription factor c-Jun (17), a component of the AP-1 transcription complex with roles in inflammation (Fig. 4*A*, *c-Jun*) (20). To determine the precise expression kinetics for this lincRNA, we performed qPCR on iBMDMs stimulated with LPS for 0, 2, 5, and 24 h (Fig. 4*B*).The lincRNA-AK170409 expression peaked at 2 and 5 h and was reduced by 24 h post-stimulation (Fig. 4*B*). lncRNAs can be found in both the nucleus and cytoplasm. We examined the cellular localization of lincRNA-AK170409 and determined that it was localized strictly to the nucleus (Fig. 4*C*). In the same manner as was used for lincRNA-Cox2, we designed two gRNAs targeting the 5′ and 3′ of the *AK170409* locus (Fig. 4*D*, *red arrows*). We electroporated gRNAs into iBMDMs, performed limited dilution isolation of clones, and screened for deletion containing clones. Using lincRNA-AK170409-specific genotyping primers (Fig. 4*D*), we confirmed deletion of one allele of the *AK70409* locus (Fig. 4, *E* and *F*), which resulted in 50% reduction in lincRNA expression, as evaluated by qPCR (Fig. 4*G*). We screened over 300 clones and failed to identify a clone with both alleles deleted suggesting that knocking out this lincRNA could be lethal to cells. Nevertheless, reduced expression of lincRNA-AK170409 resulted in a significant reduction in NF-κB signaling following stimulation with either LPS or Pam3CSK4, which suggests a role for this lincRNA in the pathway (Fig. 4, *H* and *I*). Unlike the data observed for lincRNA-Cox2, we found no difference in IκBα degradation between control and heterozygote cells (Fig. 4, *J* and *K*) suggesting that the effects of this lincRNA are on a different part of the pathway. To assess global gene expression changes that result from deletion of the *AK170409* locus, we performed Nanostring analysis on control and heterozygote iBMDMs stimulated for 2 and 5 h (Fig. 4, *L* and *M*, and supplemental Tables S1 and S2). Interestingly, we identified genes that were both up- and down-regulated when lincRNA-AK170409 was knocked down, including genes with known roles in the TLR-signaling pathway (Fig. 4, *L* and *M*, and supplemental Tables S1 and S2). Intriguingly, one of the most down-regulated genes was the aryl hydrocarbon receptor (*Ahr*) gene, which encodes a ligand-activated transcription factor known to regulate immune responses (discussed below) (21). Genes affected by knockdown of lincRNA-AK170409 were highly correlated between the 2- and 5-h time points (Fig. 4*N*). Together, by using our NF-κB–Cas9 system, we have identified two new lincRNAs that control NF-κB signaling, and we demonstrated that this system is a versatile tool for coding and non-coding gene interrogation.

## Discussion

Here, we developed a new fluorescence-based NF-κB reporter system in iBMDMs. We have enabled CRISPR/Cas9 capabilities and demonstrated proof-of-concept utility of the system for screening novel regulators of the NF-κB pathway. Furthermore, we utilized the system to identify two novel lncRNAs that regulate the NF-κB pathway.

We have identified lincRNA-Cox2 and lincRNA-AK170409 as two novel regulators of NF-κB. We have previously identified lincRNA-Cox2 as a critical regulator of immune response genes (16). Knocking down lincRNA-Cox2 resulted in reduced expression of proinflammatory genes such as *Il6*, whereas interferon-stimulated genes (ISG) were up-regulated (16). lincRNA-Cox2 is expressed in both the nucleus and cytoplasm and had previously been shown to regulate ISGs through its interaction with the nuclearly-localized Hnrnpa2/b1 and a/b proteins (16). Until now, we did not understand what factors were involved in lincRNA-Cox2's regulation of Il6. From our previous studies we knew lincRNA-Cox2 is directly induced by NF-κB binding, which also activates Il6 (16). It was unclear whether lincRNA-Cox2 was involved in positive/negative feedback to impact NF-κB activity. Here, we show that lincRNA-Cox2 deletion directly reduces NF-κB activity, which suggests a novel role for lincRNA-Cox2 as an NF-κB cofactor required for maximal activity. A recent study by Hu *et al.* (22) demonstrated that lincRNA-Cox2 mediates activation of late-primary response genes by facilitating recruitment of NF-κB subunits to the SWI/SNF chromatin-remodeling complex. This study reported no global inhibition of NF-κB activity in lincRNA-Cox2-depleted cells, demonstrating no change in IκBα degradation or p65 translocation (22). Like Hu *et al.* (22), we find that although NF-κB is reduced in our reporter system, not all NF-κB downstream genes are affected by knocking out lincRNA-Cox2. For example, we confirm that Il6 is reduced in our cells following Cas9 removal of lincRNA-Cox2; however, Tnf expression remains intact. Interestingly, Il6 requires remodeling by the SWI/SNF complex, whereas Tnf, which is an immediate early gene, does not. In contrast to the Hu *et al.* (22) study, we observed a dramatic decrease in IκBα degradation in the cytoplasm of lincRNA-Cox2 knock-out cells following LPS stimulation. This observation was especially intriguing because we do not see all NF-κB-dependent genes down-regulated. We hypothesize the low level of IκBα degradation observed in the lincRNA-Cox KO cells may still allow normal Tnf induction at the 2-h time point. These data provide the first insight into a possible role for lincRNA-Cox2 in the cytoplasm. lincRNA-Cox2 is expressed at basal levels in cells and is highly expressed at 5 h post-LPS stimulation. Because the defect in IκBα degradation is occurring very early (0.5 h) post-stimulation, we believe basal expression of lincRNA-Cox2 must be important for controlling the pathway. Although the exact mechanism of how lincRNA-Cox2 regulates Il6 remains to be determined, its clear that lincRNA-Cox2 promotes degradation of IκBα, which likely contributes to optimal activation of Il6. Further studies will be needed to obtain a more complete understanding of all the complex parts that contribute to NF-κB signaling.

lincRNA-AK170409 is a previously uncharacterized lincRNA. We identified it from RNA-sequencing data as a highly up-regulated lncRNA in iBMDMs following LPS and Pam3CSK4 stimulation. Kinetic expression analysis revealed that this lincRNA is induced as early as 2 h post-stimulation and subsides by 24 h, which is a gene expression pattern characteristic of inflammatory related genes. lncRNAs have been demonstrated to have roles in both the nucleus and cytoplasm. As lincRNA-AK170409 is strictly nuclearly localized, this suggests its modulation of NF-κB activity is likely occurring in the nucleus. We directly assessed changes in gene expression as a result of an ∼50% decrease in lincRNA-AK170409 expression. We identified genes with known roles in TLR signaling, including *Irak2* and *Tirap*. One of the genes most drastically down-regulated (∼100-fold) in lincRNA-AK170409 knockdown cells is the aryl hydrocarbon receptor (*Ahr*) gene, which encodes a ligand-activated transcription factor known to function in concert with NF-κB to regulate immune response genes (23). Expression of Ahr expression requires binding of the NF-κB subunit, RelA (p65) (21), and it has been shown to modulate the expression of genes, including Il6 in the context of lung cancer (24). Interestingly, the activation of Ahr mirrors that of p65 in that Ahr is normally retained in the cytoplasm and relocates to the nucleus upon specific stimulants (25). Stimulants that activate Ahr include numerous hydrocarbon-based air pollutants, which are implicated in promoting low-grade chronic inflammation in the lungs (26). In conclusion, these data demonstrate that lincRNA-AK170409 is a regulator of NF-κB in iBMDMs. Further studies will be needed to determine the exact mechanism by which this lincRNA regulates the numerous inflammatory related genes identified in this study.

There are currently a wide variety of NF-κB reporters commercially available, including luciferase-based assays and secreted alkaline phosphatase reporter assays, which are reconstituted in a variety of cell types. A recent study described a p65-EGFP fusion and *Tnf* promoter reporter systems in mouse macrophages, highlighting the tremendous advantage of using reporter systems to dissect complex immunological pathways (13). Here we combine a robust NF-κB–GFP reporter system with CRISPR/Cas9 capabilities into macrophages, an important cell type in the inflammatory immune response. Our reporter system is fully compatible and was intentionally designed for use in high-throughput FACS-based CRISPR screens. Hence, unlike the p65-GFP and *Tnf* reporter systems that require microscopy-based screening methods, our system opens the possibility of performing FCS-based pooled screens on tens of thousands of genes simultaneously similar to published screens in other cell lines (27). Furthermore, our NF-κB–Cas9 iBMDM system offers the ability for functional dissection of pathways involved in the “priming” steps of the inflammatory response from the downstream inflammasome program. Macrophages are critical effectors of the inflammatory response necessary for eliminating a variety of pathogens and maintaining homeostasis. Paradoxically, macrophages are also the cell type of choice for infection and replication of many distinct bacteria and viruses (28). Our system offers the ability to explore genes involved in pathogen replication, with the ability to dissect pathways specifically involved in NF-κB activation. In conclusion, we have generated a new NF-κB–GFP iBMDM reporter line, as well as a line stably expressing active Cas9. These cells will be useful for researchers interested in conducting pooled based screening, which could be used to identify novel genes involved in regulating NF-κB in response to infection or could be used for drug discovery screening. Here we utilized the system to identify lincRNA-Cox2 and lincRNA-AK170409 as novel regulators of NF-κB.

## Experimental procedures

### Cell culture and stimulation

Bone marrow cells from wild-type mice were cultured in DMEM with 10% fetal bovine serum, 100 units/ml penicillin, 100 μg/ml streptomycin (Sigma), and 20% L929 supernatants to generate BMDMs. BMDMs were immortalized with J2 virus to generate iBMDMs. Cells were stimulated for the indicated times with LPS (Tlr4) (100 ng/ml), Pam3CSK4 (Tlr2/1) (100 nm), Pam2CSK4 (Tlr2/6) (100 ng/ml), and poly(I:C) (Tlr3) (25 μg/ml) (Sigma).

### Plasmids and cloning

The NF-κB reporter construct was made by cloning five NF-κB-binding sites into the pLSmP vector via PvuII/SbfI as described previously (29). The Cas9 construct was constructed from a pSico lentiviral backbone with an EF1a promoter expressing T2A flanked genes: blastocidin-resistant (blast), blue fluorescent protein, and humanized *Streptococcus pyogenes* Cas9 (Fig. 2*A*). The gRNA construct was also constructed from a pSico lentiviral backbone driven by an EF1a promoter expressing T2A flanked genes: puro and cherry. gRNAs were expressed from a mouse U6 promoter. 20-Nucleotide forward/reverse gRNA oligonucleotides was annealed and cloned via the AarI site.

### NF-κB-CRISPR/Cas9 cell line construction and validation

iBMDMs were infected with the NF-κB reporter construct and clonally selected to maximize NF-κB–GFP induction. These cells were lentivirally infected with the Cas9 construct (described above) and were selected by blastocidin for >2 weeks to obtain Cas9-expressing cells (validated by GFP knockdown). These Cas9-expressing cells were then infected with either *Tlr4* or non-targeting gRNA-expressing virus and were puromycin selected for 1 week, before assaying effects on the NF-κB reporter. *Tlr4* gRNA sequences and lincRNA-AK170409 and lincRNA-Cox2 targeting gRNAs are also shown in Table 1.

**Table 1:** **gRNAs**

Name	Sequence
NT (non-targeting)	GTCCATACGCATAATCACCG
Tlr4-g1	TCTCTAGAAAGCTTCCCTAT
Tlr4-g2	AAAATATGCAGGTAACTTAC
Tlr4-g3	TCTGACGAACCTAGTACATG
lincRNA-Cox2-dnstrm-g1	ATCATTAACCTGTTATCATA
lincRNA-Cox2-dnstrm-g2	CTTCAATAGACATATCTTTA
lincRNA-Cox2-upstrm-g1	TCTTTGATGCAAGGAACTAC
lincRNA-Cox2-upstrm-g2	TTACACTGTTTATCGCTGGT
lincRNA-AK170409-dnstrm_g1	AGAGTATCTTCTCATAAGTA
lincRNA-AK170409-dnstrm_g2	GCAGGAGTGATTGTATGAAG
lincRNA-AK170409-upstrm_g1	GAAAAATTGGGTGTCTGGTT
lincRNA-AK170409-upstrm_g2	TTGACTTTAGATGTCGGTAA

### Cell extracts and Western blottings

Cell lysates were prepared in High Stringency Lysis buffer (50 mm HEPES, pH 7.5, 100 mm NaCl, 1 mm EDTA, 1% Nonidet P-40, 10% glycerol) containing protease inhibitor mixture (Roche Applied Science) and quantified by the bicinchoninic acid (BCA) assay (Thermo Fisher Scientific). Where indicated, the NE-PER kit (Thermo Fisher Scientific) was used for cellular fractionation prior to Western blotting or analysis by RT-PCR. Equivalent masses (∼15 μg) of each sample were resolved by SDS-PAGE and transferred to a polyvinylidene difluoride (PVDF) membrane and Western-blotted with IκBα (1:1,000; Cell Signaling). Horseradish peroxidase-conjugated β-actin monoclonal antibody (1:5,000, Santa Cruz Biotechnology) was used as loading control. HRP-conjugated goat anti-mouse (1:10,000, Bio-Rad) or anti-rabbit (1:10,000, Bio-Rad) secondary antibodies were used. ImageJ (http://rsbweb.nih.gov/ij/) was used for quantification of Western blottings.

### RNA extraction, real-time PCR, and Nanostring analysis

iBMDMs (2e6 cells/condition) were stimulated for the indicated number of hours. RNA was extracted using the Direct-zol^TM^ RNA MiniPrep kit (Zymo Research), and 1 mg of total or nuclear RNA was used for cDNA synthesis. RNA was extracted from infected BMDMs at the indicated time points using the iScript Select cDNA synthesis kit (Bio-Rad), and quantitative RT-PCR analysis was performed with the primers listed in the tables. iBMDMs were stimulated with LPS, in triplicate, for 2 or 5 h. RNA was extracted as described above, and 100 ng of total RNA was hybridized to the nCounter Mouse Immunology Gene Expression Codeset per the manufacturer's instructions and then quantified on an nCounter machine (Nanostring). Differential expression was determined using standard DEseq2 analysis. RT-PCR primer sequences are in Table 2.

**Table 2:** **qPCR primers** F is forward; R is reverse.

Name	Sequence
Il6 F	AACGATGATGCACTTGCAGA
Il6 R	GAGCATTGGAAATTGGGGTA
Ccl5 (RANTES) F	GCCCACGTCAAGGAGTATTTC
Ccl5 (RANTES) R	ACACACTTGGCGGTTCCTTC
Tnf F	CAGTTCTATGGCCCAGACCCT
Tnf R	CGGACTCCGCAAAGTCTAAG
Gapdh F	CCAATGTGTCCGTCGTGGATCT
Gapdh R	GTTGAAGTCGCAGGAGACAACC
linc-AK 170409-F	TTGCCACCATTTGAAAAACA
linc-AK 170409-R	AAGTGCCCCTTGGTTATTCC
linc-Cox-F	AAGGAAGCTTGGCGTTGTGA
linc-Cox-R	GAGAGGTGAGGAGTCTTATG
Actin (*Actb*)-F	TTGAACATGGCATTGTTACCA
Actin (*Actb*)-R	TGGCATAGAGGTCTTTACGGA
*Neat1*-F	TTGGGACAGTGGACGTGTGG
*Neat1*-R	TCAAGTGCCAGCAGACAGCA

### Genotyping

Genomic DNA was extracted from iBMDMs (1e6 cells) using Chelex supplemented with Tween 20 (Thermo Fisher Scientific) and proteinase K (Thermo Fisher Scientific) and incubated at 50 °C for 1 h. PCR was run with 1 μl of the Chelex mix, genotyping primers (0.5 μm), and 2× KAPA-TAQ master mix (KAPA Biosystems) using the standard PCR protocol. Indicated PCR product (1F/R) was gel-purified and topo-cloned (Thermo Fisher Scientific) to allow for standard Sanger sequencing. Genotyping primer sequences are in Table 3.

**Table 3:** **Genotyping primers** F is forward; R is reverse.

Name	Sequence
linc-AK170409-exon F (2F)	TTGCCACCATTTGAAAAACA
linc-AK170409-exon R (2R)	AAGTGCCCCTTGGTTATTCC
linc-AK170409-dnstrm F (1F)	TGTTGGGGGAAAGAAACACT
linc-AK170409-upstrm R (1R)	TGGAGCTTTCTTGTTTACCTGAC
lincRNA-Cox2-exon F	TCCTTTCCCCCTCAATTCTT
lincRNA-Cox2-exon R	TTTTCCCAATCTGCTTTGGT

### Statistics

Statistical significance of differences across two experimental groups was calculated using unpaired, two-tailed Student's *t* test in GraphPad Prism 6.0 software.

## Author contributions

S. Covarrubias and S. Carpenter conceived and coordinated the study and wrote the paper. E. K. R., S. Carpenter, and S. Covarrubias performed FACS-based experiments throughout the paper. S. K. processed the nanostring data by DEseq analysis. B. S. and E. K. R. performed Western blot analysis. N. H. and N. F. performed the limited dilution screening that led to the identification of the AK170409 heterozygote clone. S. Covarrubias, S. Carpenter, A. V. and E. K. R. provided technical assistance and contributed to the preparation of the manuscript and figures. M. T. M. provided critical reagents and suggestions. All authors reviewed the results and approved the final version of the manuscript.

## Supplementary Material

Supplemental Data

## Footnotes

2 The abbreviations used are:

lncRNA: long non-coding RNA
iBMDM: immortalized murine bone marrow–derived macrophage
BMDM: bone marrow–derived macrophage
lincRNA: long intergenic non-coding RNA
TLR: toll-like receptor
IκB: inhibitor of NF-κB
qPCR: quantitative PCR
ISG: interferon-stimulated gene
NT: non-targeting
gRNA: guide RNA
Ctl: control.
