# Supplementary material for: CRISPR/Cas-based screening of long non-coding RNAs (lncRNAs) in macrophages with an NF-κB reporter
Source: J Biol Chem. 2017 Oct 19;292(51):20911–20. doi: 10.1074/jbc.M117.799155 (PMC5743067; doi:10.1074/jbc.M117.799155)
Supplement: Supplemental Data [file supp_292_51_20911__index.html]

CRISPR/Cas9-based screening of long noncoding RNAs (lncRNAs) in macrophages with an NF-kappa B reporter — CRISPR/Cas-based screening of long non-coding RNAs (lncRNAs) in macrophages with an NF-κB reporter — CRISPR/Cas-based screening of lncRNAs in NF-κB macrophages — Supplemental Data 

# CRISPR/Cas-based screening of long non-coding RNAs (lncRNAs) in macrophages with an NF-κB reporter

## Supplemental Data

- Supplemental Table 2 (.txt, 17 KB) - DEseq data from 2h LPS stimulated iBMDMs (WT vs AK170409-Het)
- Supplemental Table 1 (.txt, 19 KB) - DEseq data from 5h LPS stimulated iBMDMs (WT vs AK170409-Het)
- Supplemental Figures (.pdf, 1.7 MB) - Supplemental Figures S1 and S2.
