# Supplementary material for: CRISPR/Cas-based screening of long non-coding RNAs (lncRNAs) in macrophages with an NF-κB reporter
Source: J Biol Chem. 2017 Oct 19;292(51):20911–20. doi: 10.1074/jbc.M117.799155 (PMC5743067; doi:10.1074/jbc.M117.799155)
Supplement: Supplemental Data [file 10.1074_M117.799155_jbc.M117.799155-3.pdf]

Figure S1

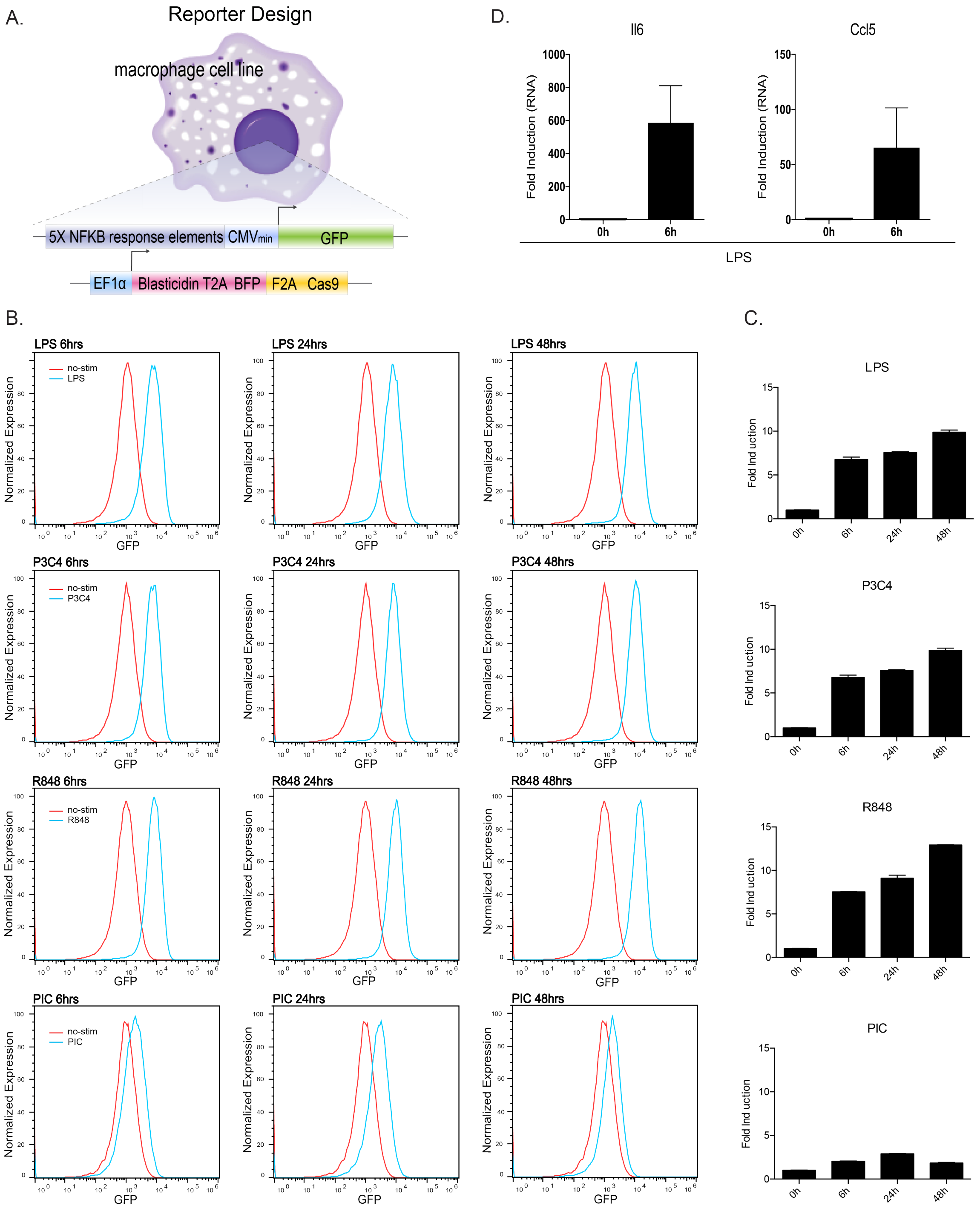

Figure S1. Addition of the CRISPR/Cas9 system into NF-KB-GFP reporter macrophages. A. NF-KB-GFP reporter cells were infected with Cas9 lentivirus and selected with blasticidin to obtain Cas9 functional cells. B. iBMDMs were either not stimulated (*red*) or stimulated with

LPS (Tlr4), Pam3CSK4 (Tlr1/2), R848 (Tlr7/8) or PolyI:C (Tlr3) ligands (*blue*) for 0, 6, 24, or 48 hours (columns 1-4). At the indicated times, cells were analyzed by FACS to assess GFP change in mean fluorescence levels. C. Histograms from B are summarized in bar format. D. Il6 and Ccl5 mRNA levels were assessed by qPCR via normalization to the house-keeping gene GAPDH. Results are presented as mean ±S.D. for at least three replicate experiments.

Figure S2

A.

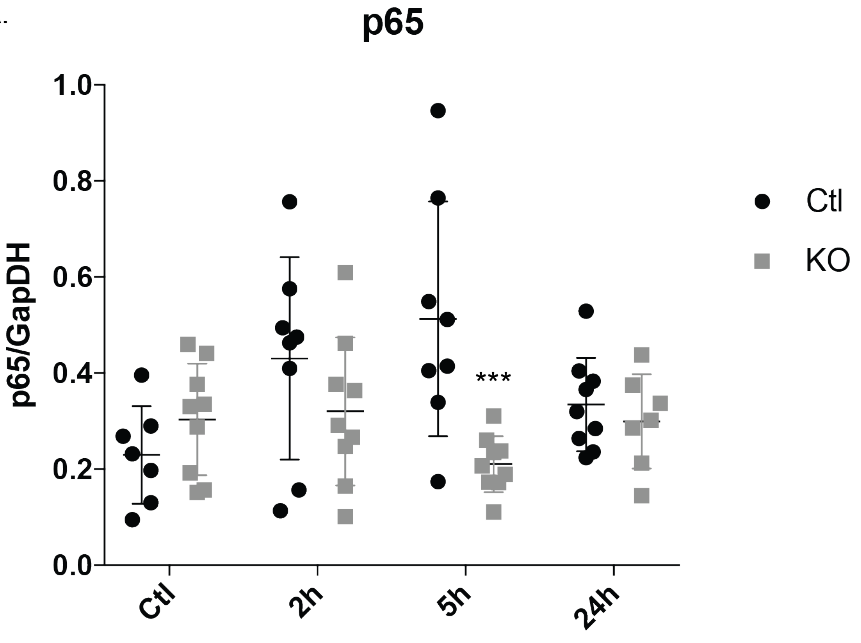

Figure S2. Time coarse of p65 expression kinetics in lincRNA-Cox2 KO Cells.

Ctl or lincRNA-Cox2 KO iBMDMs were stimulated with LPS for indicated hours and RNA was harvested for qPCR analysis. Data represents 3 independent experiments performed in triplicate. All qPCR data was normalized to the house-keeping gene GAPDH. *** $p < 0.005$.
